# Supplementary material for: Splice-Junction-Based Mapping of Alternative Isoforms in the Human Proteome
Source: Cell Rep. Author manuscript; Available in PMC 2020 Jan 15. (PMC6961840; doi:10.1016/j.celrep.2019.11.026)
Supplement: 2 [file NIHMS1546469-supplement-2.zip › DF1/PXD010154/Lung/Lung_32_RBBP4_GPDAVEERVINEEYK.pdf]

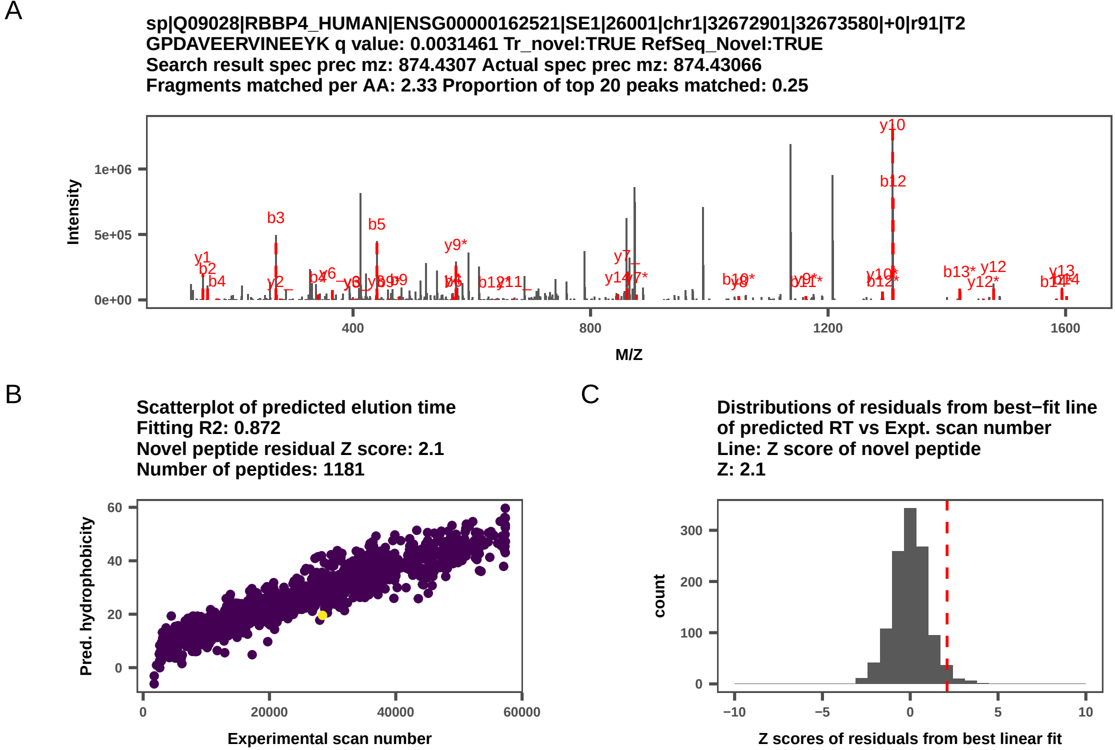
A
sp|Q09028|RBBP4_HUMAN|ENSG00000162521|SE1|26001|chr1|32672901|32673580|+0|r91|T2
GPDAVEERVINEEYK q value: 0.0031461 Tr_novel:TRUE RefSeq_Novel:TRUE
Search result spec prec mz: 874.4307 Actual spec prec mz: 874.43066
Fragments matched per AA: 2.33 Proportion of top 20 peaks matched: 0.25
Intensity
1e+06
5e+05
0e+00
y10
b12
b3
b5
y9*
y1
b2
b4
y13
b13*
y12
y12*
M/Z
400
800
1200
1600
B
Scatterplot of predicted elution time
Fitting R2: 0.872
Novel peptide residual Z score: 2.1
Number of peptides: 1181
Pred. hydrophobicity
60
40
20
0
0
20000
40000
60000
Experimental scan number
C
Distributions of residuals from best−fit line
of predicted RT vs Expt. scan number
Line: Z score of novel peptide
Z: 2.1
count
300
200
100
0
−10
−5
0
5
10
Z scores of residuals from best linear fit
